# Sex differences in dizziness diagnoses across acute and chronic neurological settings

**DOI:** 10.1007/s10072-025-08085-y

**Published:** 2025-03-08

**Authors:** Edoardo Schifino, Lucia Joffily, Nehzat Koohi, Diego Kaski

**Affiliations:** 1 Department of Biomedical Sciences, Humanitas University, Pieve Emanuele, Pavia, Italy; 2 ENT Department, Universidade Federal Do Estado Do Rio de Janeiro (HUGG -UNIRIO), Rio de Janiero, Brazil; 3 Neurology Department, Universidade Federal Do Rio de Janeiro (HUCFF -UFRJ), Rio de Janeiro, Brazil; 4 Department of Clinical and Movement Neurosciences, University College London, London, UK; 5 Ear Institute, University College London, London, UK

**Keywords:** Gender, Sex, Vertigo, Vestibular, Diagnosis

## Abstract

**Background & Objective:**

Dizziness is commoner in females and therefore clinical diagnostic frameworks are perhaps biased towards this gender. This study specifically aimed to analyse the distribution of diagnoses in neuro-otology clinics based on sex, and across age.

**Methods:**

Retrospective cohort study based on a case note review of 474 adult patients (≥ 18 years) using electronic healthcare records from patients who were referred with a primary complaint of dizziness, vertigo, or unsteadiness in outpatient neurological clinics from January 2023 to September 2024 at University College London Hospitals, UK.

**Results:**

Among the 474 patients, the most common diagnosis for dizziness was persistent postural perceptual dizziness (PPPD) (24.3%), followed by vestibular migraine (VM) (22.4%). In women the most common diagnosis was VM (31.9%), while in men it was PPPD (21.7%). In the population under 65 years old the major cause was VM (28.9%), followed by PPPD (28.6%) and “other central causes” (12.19%), while in the population over 65 years old they were benign paroxysmal positional vertigo (BPPV) (18.4%) and “other central causes” (18.4%). PPPD (28.3%) and VM (21.4%) were the commonest causes of chronic vertigo, while in the acute phase the commonest causes were VM (26%), vascular (14%) and BPPV (14%).

**Discussion:**

Overall, PPPD is the most common cause of dizziness in males, and vascular vertigo was the most common cause of acute dizziness in males, regardless of age. The combination of age and sex may be helpful in constructing *a priori* diagnostic possibilities for Neurologists, Otorhinolaryngologists and other clinicians seeing dizzy patients.

## Introduction

Dizziness and vertigo are among the most frequent complaints of patients in primary care, affecting 15–40% of the general population at some point in their lives [1, 2]. These symptoms are associated with physical and psychological disability and morbidity [3] but this can be mitigated through a correct and expedient diagnosis and appropriate management [4]. Managing patients with dizziness can be particularly challenging, as it is a vague symptom that patients may struggle to define, and can have several different causes [5–7]. Various studies have shown that patients who visited the emergency department with dizziness often left without a clear or correct diagnosis [8, 9]. Furthermore, patients with a primary complaint of dizziness, vertigo, or balance disorders must wait several years before a correct diagnosis is made and subsequent treatment is offered, principally due to non-specialist misdiagnosis [10]. A common and useful approach to diagnosing dizziness is consider the more frequent diagnoses and assess whether the clinical symptoms and examination fit with this [11]. Therefore, to improve the diagnostic process, it may be helpful to consider age-specific and sex-specific characteristics of dizziness and vertigo. It is well established that women are more to report dizziness likely than men [3, 12]. Female sex has been linked to multiple vestibular disorders such as vestibular migraine (VM), benign paroxysmal positional vertigo (BPPV) and Meniere’s Disease (MD), as well as stronger association with anxiety and avoidance behaviours [13–15]. These associations may relate to hormonal factors [16], as well as anatomic and physiological sex differences [17, 18]. However, few studies have focused on vestibular disorders in men, and while this has been attributed to a reporting bias in women, there is no data that supports this claim [17]. The aim of the study is to probe data from a neurological vestibular clinic focussing on sex differences, across age and for both acute and chronic presentations.

## Methods

### Subjects

We carried out a case note review of all adult patients (≥ 18 years old) using electronic healthcare records from patients who were referred to a vestibular neurology clinic at University College London Hospitals, UK from January 2023 to September 2024. The clinic is led by an expert neurotologist (DK) based on the Neurology Department, with a waiting list time of approximately 12 months for new patients [10]. Most patients were referred from ENT, Neurology, General Practitioner (GP), and Audiovestibular Medicine specialist services. We also reviewed notes from patients referred to an acute vertigo clinic also run by the senior author, receiving patients within 2 weeks of symptom onset, mostly referred from the Emergency Department or Stroke unit at University College London Hospitals, UK. The study included a total of 474 patients, 100 of them from the acute vertigo clinic. Ethical approval was given by the local committee.

### Diagnostic grouping

Both the final diagnosis and the data collection were conducted by Consultant Neurotologist with more than 10 years of experience and according to Barany Society classifications [19–28]. When the criteria were available, we also included patients with a probable diagnosis. We also grouped patients with diagnoses that share similarities to streamline classification based on common underlying mechanisms or affected systems. All the groups are listed below:

1. BPPV: Benign Paroxysmal Positional Vertigo
2. VN: Vestibular Neuritis
3. VM: Vestibular Migraine
4. PPPD: Persistent Postural Perceptual Dizziness (patients with a diagnosis of both PPPD and VM were considered in the PPPD group)
5. “Other central causes” includes neoplasms, Wernicke’s encephalopathy, paraneoplastic syndrome, Parkinson’s disease, central positional nystagmus, epilepsy, multiple sclerosis, peripheral neuropathy, cerebral siderosis.
6. “Other peripheral causes” includes Meniere’s disease, superior semicircular canal dehiscence, bilateral vestibular loss, unilateral vestibular loss (except vestibular neuritis), vestibular schwannoma.
7. “Systemic causes” includes vitamin deficiency, dysautonomia, cardiogenic causes, anaemia.
8. “Neurogenetic/degenerative causes” include episodic ataxia, Friedreich ataxia, mitochondrial disease, CANVAS, cerebellar ataxia, myotonic dystrophy.
9. Vascular causes include ischemic events, microvascular events, strokes and moderate to severe cerebral small vessel disease (cSVD).

We divided the sample into two age groups: < 65 years and > 65 years given that age is an important risk factors for vascular disease.

### Statistical analyses

SPSS 27.0 statistical software was used for statistical analysis. Normally distributed data were analysed using the independent sample *t*-test and Brown-Forsythe test. We used chi-square (χ2) test for comparison between groups, Yates continuity correction was performed if necessary. Differences with *p* < 0.05 were considered statistically significant.

## Results

### Demographic characteristics

Out of the 474 patients there were 189 (39.9%) men and 285 (60.1%) women with a female:male ratio of 1:1.5. The mean age of the patients was 55.39 ± 17.2 (median = 57.50, age range = 18–94), with a statistical sex difference (53.93 ± 17.5 vs 57. ± 16.5 *p* = 0.021). In relation to the setting, 374 (78.9%) presented with chronic dizziness, while 100 (21.1%) with acute dizziness. There were 322 patients (67.9%) in the < 65 yrs group and 152 patients (32.1%) were > 65 years old.

### Aetiological distribution: general

Table 1 summarises the distribution of diagnoses according to age and sex in both acute and chronic settings (see Table 2 for age distributions across diagnoses).

**Table 1:** Percentages of aetiologies across acute and chronic presentations, in different age groups and sex

Sex			Causes								
			BPPV	VN	VM	PPPD	Vascular	Neurogenetic/Degenerative	Other peripheral	Other central	Systemic causes
Female	< 65 y.o	Chronic	6 (3,7% ± 1.5)	2 (1,2% ± 0.9)	**62 (37,8% ± 3.8)**	**52 (31,7% ± 3.6)**	2 (1,2% ± 0.9)	10 (6,1% ± 1.9)	5 (3,0% ± 1.3)	19 (11,6% ± 2.5)	6 (3,7% ± 1.5)
		Acute	2 (5,3% ± 3.6)	4 (10,5% ± 5)	**17 (44,7%** ± 8.1**)**	6 (15,8% ± 5.9)	2 (5,3% ± 3.6)		1 (2,6% ± 2.6)	3 (7,9% ± 4.4)	3 (7,9% ± 4.4)
	> 65 y.o	Chronic	**12 (19,0% ± 4.9)**	1 (1,6% ± 1.6)	6 (9,5%3.7)	**15 (23,8% ± 5.4)**	2 (3,2% ± 2.2)	3 (4,8% ± 2.7)	3 (4,8% ± 2.7)	**12 (19,0% ± 4.9)**	9 (14,3% ± 4.4)
		Acute	**7 (35,0% ± 10.7)**		**6 (30,0% ± 10.2)**	1 (5,0% ± 4.9)	2 (10,0% ± 6.7)			2 (10,0% ± 6.7)	2 (10,0% ± 6.7)
Male	< 65 y.o	Chronic	2 (2,3% ± 1.6)	4 (4,5% ± 2.2)	12 (13,6% ± 3.7)	**32 (36,4% ± 5.1)**		11 (12,5% ± 3.5)	7 (8,0% ± 2.9)	15 (17,0% ± 4.0)	5 (5,7% ± 2.5)
		Acute	3 (9,4% ± 5.2)	**6 (18,8% ± 6.9)**	2 (6,3% ± 4.3)	2 (6,3% ± 4.3)	**6 (18,8% ± 6.9)**	2 (6,3% ± 4.3)	4 (12,5% ± 5.8)	2 (6,3% ± 4.3)	**5 (15,6% ± 6.4)**
	> 65 y.o	Chronic	7 (11,9% ± 4.2)	2 (3,4% ± 2.4)		7 (11,9% ± 4.2)	5 (8,5% ± 3.6)	**11 (18,6% ± 5.1)**	6 (10,2% ± 3.9)	**14 (23,7% ± 5.5)**	7 (11,9% ± 4.2)
		Acute	2 (20,0% ± 12.6)	1 (10,0% ± 9.5)	1 (10,0% ± 9.5)		**4 (40,0% ± 15.5)**				2 (20,0% ± 12.6’)

*BPPV*: Benign Positional Paroxysmal Vertigo; *VN:* Vestibular neuritis; *VM:* Vestibular migraine; *PPPD:* Persistent Postural-Perceptual Dizziness

**Table 2:** Mean age of diagnosis for the different aetiologies

Causes	Mean age
BPPV	68,5 ± 13,8
VN	56,5 ± 11,5
VM	46,6 ± 14,6
PPPD	50,1 ± 16,4
Vascular	68,0 ± 13,4
Neurogenetic	59,4 ± 16,0
Other peripheral	57,7 ± 15,5
Other central	58,3 ± 17,5
Systemic causes	62,7 ± 17,3

*BPPV*: Benign Positional Paroxysmal Vertigo; *VN:* Vestibular neuritis; *VM:* Vestibular migraine; *PPPD:* Persistent Postural-Perceptual Dizziness

Persistent postural perceptual dizziness (PPPD) (24.3%) was the most common diagnosis, followed by VM (22.4%), other central causes (14.1%), BPPV (8.6%), systemic causes (8.2%), neurogenetic/degenerative causes (7.8%), other peripheral causes (5.5%), vascular causes (4.9%), vestibular neuritis (VN) (4.2%).

### Aetiological distribution: sex

**Fig. 1:**
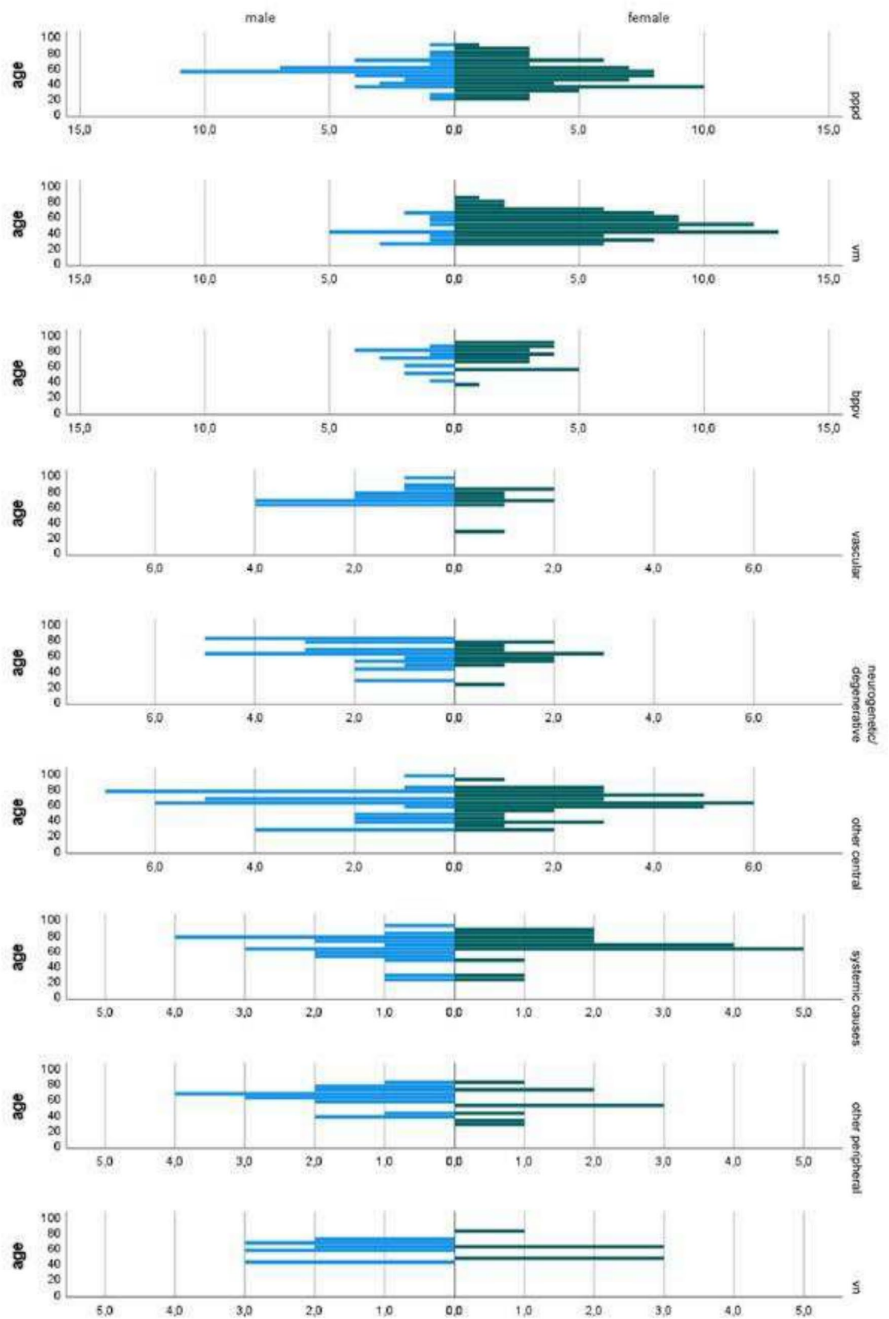
Demographic pyramid: number of patients, by age and sex, across the different aetiologies. X-axis denotes frequencies

The sex ratio was significantly different among the disorders (Chi-square test,* p* < 0.001). In VM, BPPV, and PPPD most patients were women (Fig. 1) VM showed the highest proportion of female patients (85.8%). In contrast, other peripheral causes, vascular causes, VN, neurogenetic/degenerative causes had a higher prevalence of males (Fig. 1). There was no statistical difference between sex in systemic causes (*p* = 0.24) and other central causes (*p* = 0.25). Male sex was a significant protective factor against vestibular migraine (OR = 0.2, 95% CI: 0.1–0.3), while ORs for other conditions were not statistically significant.

**Fig. 2:**
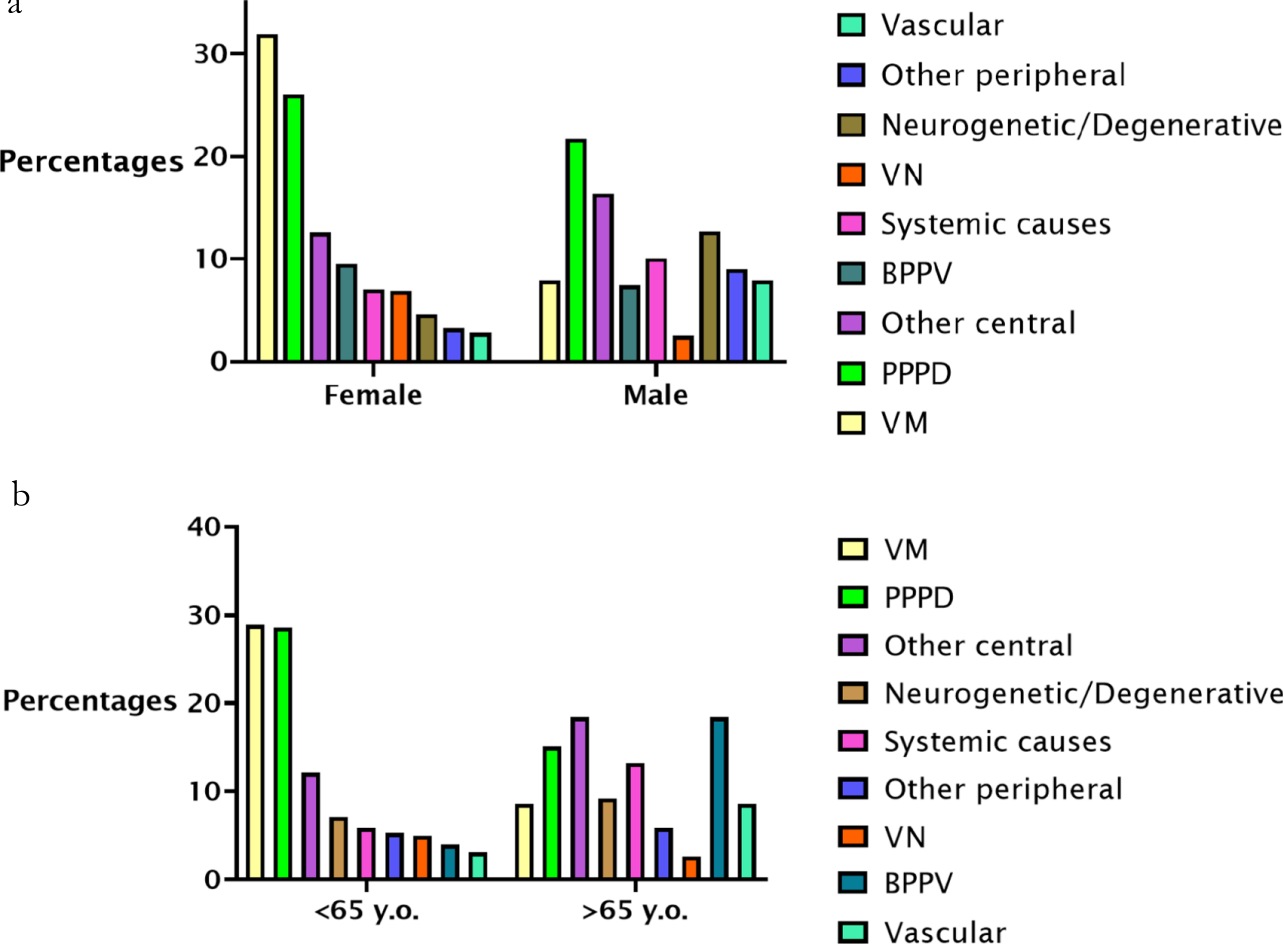
**a** Percentages of aetiologies in female and male groups. **b** Percentages of aetiologies in < 65y.o. and > 65y.o. groups

Analysing the prevalence of the various aetiologies in the female and male groups separately (Fig. 2a), we found that VM (31.9%) was the most common diagnosis in women, followed by PPPD (26%), other central (12.6%), BPPV (9.5%), systemic causes (7%), neurogenetic/degenerative causes (4.6%), other peripheral causes (3.2%), vestibular neuritis (2.5%). In men the most prevalent diagnosis was PPPD (21.7%), followed by other central causes (16.4%), neurogenetic/degenerative causes (12.7%), systemic causes (10.1%), other peripheral causes (9%), vascular (7.9%), VM (7.9%) BPPV (7.4%), vestibular neuritis (6.9%)).

### Aetiologic distribution: age

The age distribution was significantly different across vestibular disorders, both in relation to age itself (Brown–Forsythe test, *p* < 0.001) and age groups (Chi-square test,* p* < 0.001). In both MV and PPPD, most individuals were younger (< 50rs). On the other hand, in BPPV, vascular, other central and other systemic causes most of the patients were elderly (> 65yrs, Fig. 1).

Analysing the prevalence of the various aetiologies in the two age groups separately, we found that in the < 65 years old group the major cause was VM (28.9%), followed by PPPD (28.6%), other central causes (12.19%), neurogenetic/degenerative causes (7.1%), systemic causes (5.9%), other peripheral causes (5.3%), VN (5%), BPPV (4%), and vascular (3.1%). In the > 65 years old age group the most common diagnosis was BPPV (18.4%), other central causes (18.4%), PPPD (15.1%), systemic causes (13.2%), neurogenetic/degenerative causes (9.2%), vascular (8.6%), VM (8.6%), other peripheral causes (5.9%), VN (2.6%) (see Fig. 2b).

### Aetiological distribution: type of presentation

**Fig. 3:**
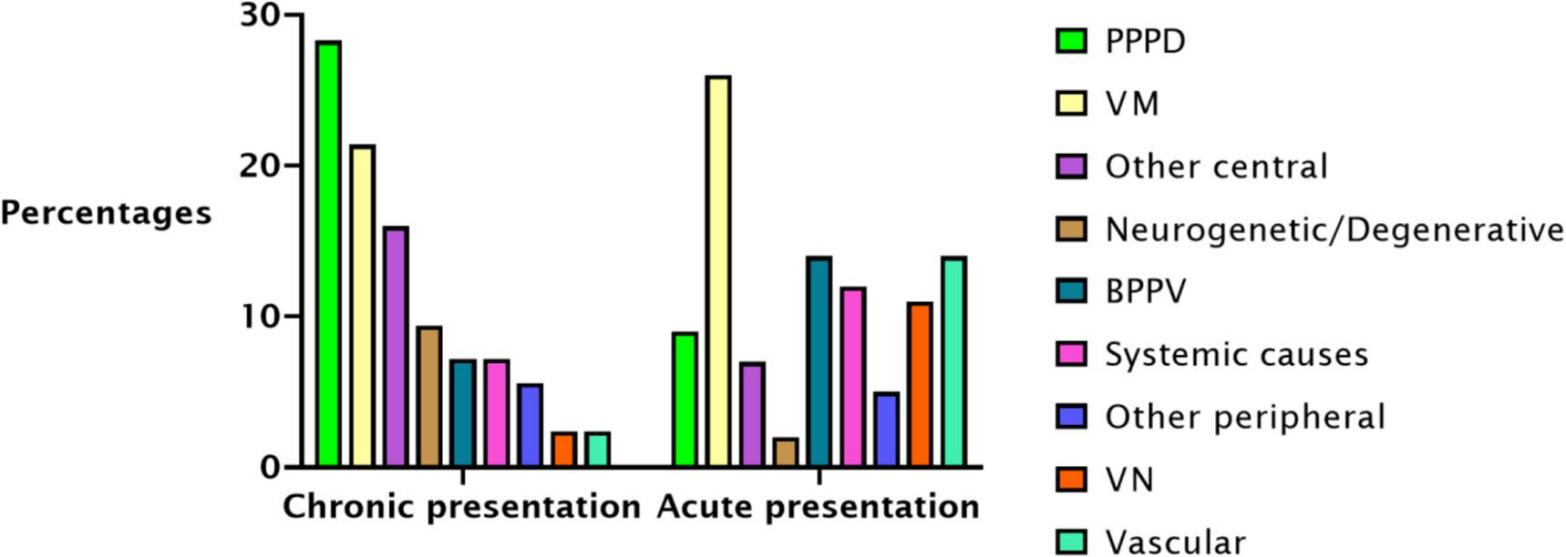
Percentages of aetiologies in acute and chronic presentations

As expected, the frequency of vestibular disorders differed in acute and chronic settings (Chi-square test,* p* < 0.001*)*. Neurogenetic/degenerative, PPPD, other central causes, other peripheral causes, VM, systemic causes, BPPV, were more prevalent in the chronic settings. VN and vascular causes were instead more frequently presented acutely. The most common cause in chronic presentations was PPPD (28.3%), followed by VM (21.4%), other central causes (16%), BPPV (7.2%), neurogenetic (9.4%), systemic causes (7.2%), other peripheral causes (5.6%), VN (2.4%), vascular (2.4%). VM (26%) was the most prevalent cause presented acutely, followed by vascular (14%), BPPV (14%), systemic causes (12%), VN (11%), PPPD (9%), other central causes (7%), other peripheral causes (5%), neurogenetic (2%) (see Fig. 3).

## Discussion

We conducted a retrospective single-centre study reviewing case notes of adult patients referred with dizziness, vertigo, or unsteadiness to specialist vestibular neurology outpatient clinics and an acute vertigo service. We provide the most common diagnosis in male and female patients presenting with dizziness within this neurological setting, to guide the diagnostic process.

PPPD and VM were by far the most common causes in our population sample, as has also been reported in other epidemiological studies [29]. In contrast to the findings of these studies, the statistical representation of BPPV and other peripheral disorders was relatively low in our analysis. This inconsistency may be due to our clinic being a highly specialized, referral-based centre that receives fewer patients with conditions treatable in emergency departments or primary care settings. Moreover, our unit is a Neurological tertiary referral centre and therefore neurological presentations may be more common than ‘otological’ ones, making our findings more relevant to a Neurological audience rather than an otolaryngology setting. Finally, our cohort included more individuals under the age of 65 years than those over this age, perhaps contributing to the lower number of BPPV cases than in other reported cohorts.

### Sex

We showed that patients with VM, BPPV and PPPD are more commonly female (Fig. 1). There is indeed extensive evidence supporting a strong association between female sex and both VM and BPPV, which has been linked to a correlation between hormonal profiles [16, 17, 30]. It has also been reported that VM has genetic predisposition displaying autosomal dominant inheritance with reduced penetrance in males [31]. While there is limited epidemiological data available for PPPD, given its relatively recent classification, multiple studies have already shown a higher prevalence in women [17, 29]. However, unlike VM and BPPV, higher incidence of PPPD in women may be less linked to hormonal influences relating directly to oestrogen [17].

Conversely, neurogenetic, other peripheral, vascular vertigo, and VN were more common in men (Fig. 1). Vascular vertigo has already been shown to be more associated with male sex, and this has been linked to a higher proportion of cardiovascular risk factors in males [31]. On the other hand, there is conflicting evidence regarding the incidence of VN by sex, with some studies that report a higher proportion in men [17, 32, 33], while other studies support the opposite [14]. For specific disorders included in other peripheral causes (e.g., MD, superior semicircular canal dehiscence, vestibular schwannoma, and bilateral vestibular failure) there does not seem to be any strong sex bias [17]. Equally, the specific conditions included in our neurogenetic/degenerative causes are expected to affect males and females equally [34], so we speculate that the higher prevalence we found in men may relate to a small sample size. Neurogenetic causes of vertigo may be less prevalent in otolaryngology settings, but the recent identification of genes responsible for common vestibular ataxia syndromes (e.g., CANVAS and SCA27B) suggest these patients will be increasingly prevalent in otolaryngology clinics also.

In females, VM and PPPD accounted for more than half the total diagnoses, with the other causes being far less represented and more scattered (Fig. 2a). Conversely, in males, the main diagnoses were PPPD, other central causes, and neurogenetic/degenerative causes, followed closely by all the other diagnoses, except for vestibular neuritis. Xing and colleagues recently performed similar analysis for dizziness patients [32], but that data cannot be directly compared due to the use of different diagnostic criteria. Importantly, our findings suggest that diagnosing dizziness in males may be more challenging than in females due to a more even frequency distribution of potential causes.

### Age

Patients with VM (and PPPD) were younger (Fig. 1), in keeping with other studies reporting a mean age of onset of VM of approximately 40 years [35, 36] and in PPPD in the mid-40 s [37]. BPPV was most diagnosed in the > 65 years old age group, together with vascular, other central and systemic causes. BPPV is known to be more prevalent in older individuals, especially in women [38], and the same has been shown for vascular vertigo [39] and most of the conditions we included in the other central and other systemic diagnostic groups.

In patients under 65 years old vestibular migraine and PPPD accounted for more than two thirds of all the diagnosis, data that should prove useful when considering possible causes for a younger dizzy patient. Finally, in the population older than 65 years old, the major causes for dizziness were other central, BPPV, PPPD, and systemic causes. From a practical perspective, in the older population, the underlying cause of dizziness is more challenging, with several aetiologies sharing a similar likelihood. However, in an elderly male patient with dizziness, a vascular cause is more prevalent than BPPV (Fig. 1). This finding may differ in an ENT department, where BPPV is more prevalent, compared to a Neurology based centre like the one in the current study [10]. Additionally, since BPPV is often managed in primary care centres with repositioning manoeuvres, the number of patients presenting to tertiary care centres may be lower than what is observed in the general population.

### Acute-chronic presentation

We investigated how specific vestibular diagnoses differ between acute and chronic settings, across sex and age. As expected, only VN and vascular vertigo were more common in acute presentations than in chronic neurology outpatient clinics. Of note, we expected BPPV to be more prevalent in acute settings but given the study setting of a specialised neurological centre, some patients with BPPV may have not been referred to us due to spontaneous resolution of symptoms, referral to other less specialist services, or appropriate community-based treatment. In the acute setting, the main causes were VM, BPPV, and vascular vertigo, followed closely by vestibular neuritis and systemic causes.

In our sample we found that in dizzy female patients younger than 65 years old who present either chronically or acutely the main diagnosis to consider is VM followed by PPPD. While in women above 65 years old, chronic symptoms are more likely to be caused by PPPD, while acute symptoms by BPPV, followed by VM. Although VM is known to decrease with age, it is still the second most common cause of dizziness in the emergency department in older women. In male patients younger than 65 years old who present with chronic dizziness symptoms the main diagnoses to take into consideration are PPPD, followed by other central causes. In younger and older men with acute dizziness, vascular causes are the most prevalent, but this increases with age (Fig. 1).

We recognise some limitations of this study; first, this study was conducted in a referral-based hospital mostly run by neurologists. Thus, the selection and referral biases would have been inevitable, and the results cannot be generalised to other primary clinics or referral-based clinic mostly run by ENT doctors. Secondly, we report on relatively small sample sizes, although this in turn allowed us to have greater certainty in relation to clinical data and diagnoses.

## Conclusion

Although we caution against defining dizziness aetiology using only demographic data and presentation type (acute-chronic), interesting patterns were found across the subgroups studied. In women, VM was the most prevalent aetiology, decreasing with age and occurring frequently in both chronic and acute scenarios. BPPV is more prevalent at older ages in both sexes. PPPD was the most common cause of chronic dizziness in men. Vascular vertigo was the most common cause of acute dizziness in males, regardless of age, highlighting the importance of careful assessment to identify central features. Replication of this data across otolaryngology settings would be welcome to better understand gender differences in all patients presenting with dizziness.

## Data Availability

The datasets during and/or analysed during the current study available from the corresponding author on reasonable request.
